# Dr. M. Hall's Discovery in Reference to the Functions of the Spinal Marrow

**Published:** 1837-04

**Authors:** 


### DR. MARSHALL HALL'S DISCOVERY IN REFERENCE TO THE FUNCTIONS OF THE SPINAL MARROW.

Since the publication of our last Number, we have had some correspondence with Dr. Marshall Hall, on the subject of an article contained in it, "On the Physiology of the Nervous System;" he being of opinion that justice was not done by the Reviewer to his views of *the Reflex Function of the Spinal Marrow.* Dr. Hall having kindly favoured us with his own statement of his claims, we willingly accede to his request to present this to our readers without alteration. We shall append a very few notes to Dr. Hall's letter, in illustration and (if we may say so) corroboration of certain opinions advanced in the article referred to; but no more than seems unavoidable on our part. Indeed, had not Dr. Hall's letter related to physiological questions of great importance we could not have noticed the subject again in our Journal; as we determined, from the very beginning of our labours, not to occupy its pages with matters of personal controversy, even should we have the misfortune, as on the present occasion, to differ in opinion with men of distinguished scientific and professional eminence. As, in our brief remarks on Dr. Hall's letter, we shall studiously restrict ourselves to the defence or illustration of statements already made by us, we hope this amicable controversy will here terminate.

### LETTER FROM DR. HALL TO HIS REVIEWER.

"I very gladly send you, as you kindly request, a very brief account of what it is I claim as my discovery, in reference to the functions of the spinal marrow.

"The first *fact* which I observed was that presented by the tail of the salamander, recently separated from the trunk: on being touched by the point of a needle, it coiled up with energy. This phenomenon ceased on destroying the portion of the spinal marrow within the caudal vertebræ, the irritability still remaining.

"Did I claim the discovery of this fact?—No more than Newton ("sic parvis componere magna,") claimed the discovery of the *fact* that an apple would fall if its point of suspension were removed.

"But the fact was to me as the fall of the apple. It suggested to me what I do claim—viz. the *principle*, 'novel and extensive in physiology,' on which it itself, and, 1, all the *experimental* facts noticed by Redi, Whytt, Legallois, Mr. Mayo, &c.; 2, the series of *physiological* phenomena, beginning with the act of winking on touching the eyelash, and terminating with the tonic condition of the whole muscular system; and, 3, the series of *pathological* events, beginning with the effects of teething, and including all those diseases of the nervo-muscular system which have not their source in the nervous centres,—are to be explained.

"This principle is expressed (however uncouthly) by saying that it is, in certain cases, *direct*, but far more frequently *reflex*, and always *excito-motory;* nor can I dispense with any one of these epithets.

"I may observe, that this principle is distinct from *sensation* and voluntary motion. It exists in the human subject in cases of perfect paraplegia, in which the patient can bear testimony to the fact. It must then be novel.

"It is the peculiar and special property of the medulla spinalis, and entirely independent of the brain and of the sympathetic nerve, existing when these are removed.

"The brain, the olfactory, the optic, the acoustic nerves, are destitute of the excito-motory property; facts proved by M. Flourens and M. Magendie. This property begins at the tubercula quadrigemina, and subsists in the whole spinal marrow and the motor nerves. These facts have been ascertained by Lorry, M. Flourens, Professor Müller. Its action is *direct;* that is, it pursues the course of the spinal marrow and motor nerves, *from* the nervous centre *to* the muscular system. Its principal physiological action (and this observation I claim,) is in giving *tone* to the muscular system. It is affected pathologically in all *centric* diseases of the spinal marrow, and in diseases of the motor nerves.

Prof. Müller proceeded a step further. In a most interesting paper, published in the "Annales des Sciences Naturelles," (tom. xxiii. p. 108,) he states that, on laying bare and dividing the spinal marrow in a frog, and irritating by the forceps, or by galvanism, the extremity of its upper portion, the head and anterior extremities were moved; demonstrating that the spinal marrow possesses a *retrograde* influence, and consequently is something more than a mere bundle of nerves.

"I have proceeded a step further still. 'I have found,' (*Lectures*, paragraph 57,) 'that movements of the limbs are excited by pinching the intercostal nerves, as they leave the spine, continuously, by the forceps. The influence of this stimulus is not only *reflected* upon the limbs, but it is *retrograde* in its course, passing from a nerve proceeding from the middle part of the spine *forwards* to the anterior, as well as backwards to the *posterior* extremities. This experiment appears to me to present us with the simplest *type* of some spasmodic diseases, and especially of the traumatic tetanus.'

"This last experiment I claim. I do not claim any of the facts to be found in Redi, Whytt, Legallois, or Mayo, any more than Newton claimed the universally known facts of the revolution of the planets in their orbits, or Haller the equally well-known fact of the contraction of the heart and other muscles on the application of a stimulus. But I do claim this experiment; and I do so because I think that it is new, and that it proves a "novel and extensive principle in physiology;" that it proves, in fact, the existence,

"1. Of a true Spinal Marrow, *physiologically* distinct from the cord of intra-vertebral nerves.

"2. Of a system of Excito-motory Nerves, *physiologically* distinct from the nerves of sensation and voluntary motion.

"3. Of currents of Nervous Influence, incident, upwards, downwards, and reflex with regard to the spinal marrow.

"I repeat, that this experiment affords the type, or simplest form, of the series of physiological and pathological phenomena to which I have alluded; and especially of all the diseases of the nervo-muscular system, having their origin, like dentition, traumatic tetanus, &c., in a point situated eccentrically in reference to the spinal marrow.

"I claim the division of spasmodic diseases founded upon these views, into those having their origin in incident nerves, in the spinal marrow itself, and in motor nerves; the three divisions of the excito-motory system. Approximations may have been made to this view of spasmodic diseases, but, until the system itself was understood, its pathology must have been obscure; and I do not wish to dispute with those who, like the adversaries of Lavoisier, "déterrait, pour le chagriner, tous les vieux livres ou pouvaient se trouver quelques idées analogues aux siennes." (*Eloges historiques*, par M. le Baron Cuvier, tome iii., p. 186.)

"I feel persuaded that you will not be offended with me for my frankness; I will therefore make an observation or two upon your Review.

"First, it was a mistake to say that 'it was ascertained by Legallois, Magendie, Flourens, and others, that the parts of the nervous system essentially concerned in sensation do not extend higher than the medulla oblongata.' Whatever Legallois may have thought, M. Flourens' opinion is directly the contrary.*

"Secondly, it was a mistake to add, that 'it became obvious to every one who reflected on the subject at all, that, if the doctrine of the dependence of sympathetic actions on sensations is true, it must be the spinal cord and medulla oblongata, not the brain, that must be concerned in producing that description of muscular actions.' (*B. and F. Med. Rev.*, No. V., p. 34.)

"Dr. Alison, for instance, has written upwards of sixty pages upon this subject without once mentioning either the medulla oblongata or spinal marrow.† The thing could not, therefore, be so very obvious.

* Our reference, it will be observed, was not to the work of Flourens, but to the Report, by Cuvier, to the French Institute, on the experiments of Flourens; the language of which is much more precise and accurate. Cuvier's words are, "C'est tout à fait dans le haut de la moelle allongée, à l'endroit ou les tubercules quadrijumeaux lui adherent, *que cesse cette faculté* de recevoir et de propager d'une partie de l'irritation, et de l'autre la douleur. C'est à cet endroit au moins que doivent arriver les sensations pour être percues, c'est de là au moins que doivent partir les ordres de la volonté. Ainsi la continuité de l'organe nerveux, depuis cet endroit jusqu'aux parties, est necessaire à l'éxécution des mouvemens spontanés, à la perception des impressions, soit intérieures, soit extérieures." See Journal de Physiologie, t. ii., p. 384; and he argued, as we think justly, *against* the supposition that the various movements seen in an animal, of which the cerebral hemispheres have been removed, "s'operent sans être provoquées par aucune sensation," (*ib.* p. 381:) i.e. he thought that a degree of sensation remains in an animal thus mutilated. It will be remembered that, in some of the experiments of Flourens and Bouillaud, animals lived for months, and grew fat, in which the cerebral lobes had been removed, and which merely swallowed food put into their mouths, never seeking nor seizing food; and, when not roused by irritation, appearing to be in profound sleep. Were these animals devoid of sensation? If not, how did the phenomena presented by them differ from those which are here called "excito-motory?" Is respiration (an excito-motory phenomenon,) independent of sensation? Those who think so will find many physiologists besides Whytt opposed to them.

† Dr. Alison, in his paper on the Physiological Principle of Sympathy, professes to do no more than illustrate and confirm the doctrine of Whytt on that subject. Now, Dr. Whytt's doctrine is, "that the various sympathetic motions of animals, produced by irritations, are owing to particular sensations excited in certain organs, and thence communicated to the brain *or spinal marrow;*" and, therefore, that "all sympathy must be referred to the brain *and spinal marrow*, the source of all the nerves." (*Of the Sympa-*

"I was much annoyed to find my experiment on the horse (*op. cit.* p. 34,) compared with some experiments by Whytt on the frog. What was the *gist* of my experiment? To prove that, when *sensation* was annihilated, excito-motory phenomena remained. Was this the object of Whytt's experiment? Certainly not. Whytt considered the phenomena which he observed to be dependent on sensation. Then the two experiments do not fairly admit of comparison; and certainly I am as far from being of Whytt's opinion as the poles are asunder. This physiologist concludes, 'If the motions of the muscles in a cock's limbs, after decollation, are, without doubt, owing to its *soul*(!), may we not also ascribe to the same principle the like, but less remarkable motions, in men and quadrupeds, after their heads are struck off, and, consequently, the tremulous motions and palpitations of their *hearts* (!) too, after death or separation from their bodies?' (*Whytt's Essay on the Vital Motions*, p. 389.)

"I beg, in conclusion, to call your particular attention to the preface to my "Lectures," and to the recent confirmation of my views by Prof. Müller.

"I am, &c. &c. "MARSHALL HALL."

"14, *Manchester square; Feb.* 2, 1837."

We confess that the analogy of the case of Sir Isaac Newton and the fall of the apple did not occur to us; but we gladly take this opportunity of expressing our high respect for the zeal, industry, and physiological attainments of Dr. Marshall Hall; and our regret that he should think himself unfairly treated by any criticism of ours. But, after carefully considering his explanation, (part of which we are not certain that we exactly comprehend,) we cannot see that, in justice to former authors, and particularly to Dr. Whytt, we can retract the opinion formerly expressed. The phenomena which he describes are precisely of the same class as those described by the other authors formerly quoted: those of them which are not the direct effect of the irritation of motor filaments of nerves, come strictly under the definition of sympathetic actions, and are important as illustrating the mode of production of that important class of vital actions; and the general doctrine of Whytt in regard to all such phenomena, when limited by modern discoveries in the way stated in our note on Dr. Hall's letter, and several of his individual experiments clearly and demonstrably point to the spinal cord, as the main agents in producing them. We have explicitly ascribed merit to Dr. Hall for "fixing the attention of physiologists on these actions, and on the agency of the spinal cord in producing them, and likewise for casting doubts on the doctrine of their dependence on sensation;" and have admitted that this last question is still *sub judice;* only pointing out that, in the entire and perfect animal, these actions are always preceded and attended, if not directly caused, by sensations, and are obviously intended to remove the causes of those sensations; and our readers can now judge for themselves whether a novel and important principle, overlooked by us, has been established by Dr. Hall.

*thy of the Nerves*, in Whytt's works, p. 510-11.) And Dr. Alison has expressly stated, at the conclusion of his paper, that "modern physiologists have rendered this doctrine somewhat more precise, by determining the portions of the encephalon which appear essential to the sensations, and the actions of nerves in consequence of them, taking place." (*Edinb. Medico-Chirurg. Transactions*, vol. ii., p. 223, published in 1826.) The limitation of the parts of the encephalon thus assigned to sensations, and to their effects, is thus stated in the same author's Outlines of Physiology: "It is now satisfactorily ascertained that no part of the brain, higher than the corpora quadrigemina, nor of the cerebellum, is essentially concerned in sensation." (P. 131.) Now, if we acquiesce in the doctrine of Whytt, ascribing sympathies to the brain and spinal marrow, but limit it to the extent of excluding all parts of the brain higher than the corpora quadrigemina, what remains to be concerned in them but the medulla oblongata and spinal marrow? And, if this was not expressed *totidem verbis*, was it not because the expression of it appeared really superfluous?

It is true that Dr. Whytt, in the passage quoted by Dr. Hall, and in many others, has pushed his favorite doctrine of muscular action produced by sensation to an unwarrantable length, extending it to the contractions produced by direct physical irritation of the motor nerve, even of an amputated limb, and to the usual contractions of the involuntary muscles; but his errors in these respects are foreign to the present discussion, which relates only to muscular movements produced by irritation of distant parts, and especially of the sentient extremities of distant nerves.

As to the paper of Professor Müller, "on the Reflex Function of the Spinal Marrow," kindly communicated to us by Dr. Hall, (and published in the London and Edinburgh Philosophical Magazine, for December last,) we must be permitted, with all respect for that very meritorious author, to observe, that it seems to us to prove nothing more distinctly than that the learned author, like many physiologists in this country, had not duly studied the writings of Whytt and Monro on the Nervous System. The coincidence of his views with those of Dr. Whytt is the more remarkable, as he does not, like Dr. Hall, exclude sensation from the production of the phenomena of which he treats. His doctrine is shortly and distinctly stated by himself as follows: "When *perceptions*,* which are produced by external stimuli on sensitive nerves, produce motions in other parts, this never takes place by a reciprocal action of the sensitive and motor filaments of the nerves, but by the sensorial excitement acting on the brain and spinal marrow, and from these back to the motor filaments. This extremely important principle in physiology and pathology requires a strong proof, which may be very clearly attained empirically, and then explains a number of physiological and pathological phenomena;"† and he particularly enumerates the actions of the respiratory muscles in breathing, coughing, sneezing, vomiting, the expulsion of fæces and urine, &c., as admitting only of this kind of explanation.

In the sentence above quoted, three propositions are stated: 1. That external stimuli may produce motions in other (i.e. distant) parts, by impressing sensitive nerves and exciting sensations; 2. That this fact is not to be explained by a reciprocal action of the sensitive and motor filaments of nerves, (i.e., as he afterwards explains, by the connexions or anastomoses among nerves;) and, 3. That it is to be explained by an excitement carried to the brain and spinal marrow, and thence back to the motor nerves of individual muscles.

Now, that there is nothing in these propositions which was not previously familiar, at least to some British physiologists, and which was not regarded by them as established by the labours of physiologists of the last century, we think we shall unequivocally demonstrate by three short quotations from Dr. Alison's paper on the Physiological Principle of Sympathy, already mentioned.

"In those sympathetic actions in which the organs of animal life are concerned, the phenomenon observed is, that, on an irritation or stimulus being applied to one part of the body, the voluntary muscles of another and often distant part are thrown into action; as when the respiratory muscles are excited, apparently by changes in the lungs, in ordinary breathing, or by irritation of the nostrils or trachea in sneezing or coughing; the distant muscles concerned in vomiting, by irritations acting on the stomach; the distant muscles concerned in the expulsion of the fæces and urine, by irritation of the rectum and bladder, &c." (*Ed. Med.-Chir. Transactions*, vol. ii. p. 169.) "The researches of Dr. Whytt and Dr. Monro were, I think, quite successful in establishing two points in regard to such phenomena: 1. That they cannot be explained by the connexions of the nerves of the sympathising parts; and, 2. That they

* This should have been, we think, translated "*sensations*:" in the original, *Empfindungen*.—Rev.

† Extract from Prof. Müller, by Dr. Hall, p. 8.

do not indicate any necessary consent or sympathy *between individual parts* of the body, but are, in general, simply the effects of certain mental *sensations;* and that, in these instances, one part of the body sympathises with another only in so far as the sensation which is the natural and appropriate stimulus of the one, is excitable by irritation of the other." (*Ib.* p. 170.) "We have seen evidence sufficient, as I think, to induce us to acquiesce in the proposition that these actions must generally depend on the excitation of particular sensations. In order that these sensations may be felt, the nerves, from the impressions on which they proceed, must be entire up to the brain; but, when they are strongly felt, their influence extends, or is *reflected* downwards, often to parts of the nervous system remote from those in which they originated. Each acts as a stimulus, or excites an involuntary instinctive impulse, which acts as a stimulus, on particular muscles only; and we cannot tell why. *This was the doctrine of Whytt, of Monro, and of Haller.* Modern physiologists have rendered it more precise, only by determining the portions of the encephalon which appear essential to the sensations, &c. (as quoted in the note above.) Further than this, in the explanation of sympathetic actions, I cannot go; and I will venture to maintain that, whoever does go further, goes on hypothesis, and will find himself opposed by facts." (*Ib.* p. 222.)

In order to perceive the exact coincidence of the "extremely important principle" stated by Müller with the doctrine thus previously held to be established, it is only necessary to add, that the distinction of sensitive and motor nerves, laid down by Sir Charles Bell, is distinctly admitted and recognized in the paper in question, (see p. 191;) so that the transmission of the sensations upwards must necessarily be ascribed to the sensitive filaments of the nerves, and that of the stimulus thence resulting downwards, to the motor filaments, as stated by Müller.

